# Complete mitochondrial genome sequence of *Acromitus flagellatus* and its phylogenetic relationship with related jellyfish species

**DOI:** 10.1080/23802359.2022.2131367

**Published:** 2022-10-21

**Authors:** Jianing Lin, Song Feng, Lijuan Wang, Yanhao Qiu

**Affiliations:** a Institute of Eco-Environmental Forensics, Shandong University, Qingdao, China; b School of Environmental Science and Engineering, Shandong University, Qingdao, China; c CAS Key Laboratory of Marine Ecology and Environmental Sciences, Institute of Oceanology, Chinese Academy of Sciences, Qingdao, China; d Laboratory for Marine Ecology and Environmental Science, Pilot National Laboratory for Marine Science and Technology (Qingdao), Qingdao, China; e Center for Ocean Mega-Science, Chinese Academy of Sciences, Qingdao, China; f College of Marine Science, University of Chinese Academy of Sciences, Qingdao, China

**Keywords:** *Acromitus flagellatus*, scyphozoan, jellyfish blooms, mitochondrial genome

## Abstract

This study describes the complete mitochondrial genome sequence of the scyphozoan *Acromitus flagellatus* (Maas, 1903), a blooming jellyfish found in the coastal areas of Hainan, China. Its mitochondrial DNA is 16,779 bp in length and has a linear structure, comprising 13 protein-coding genes (PCGs), two rRNAs (s-rRNA and l-rRNA), and two tRNAs (trna-W-TCA and trna-M-CAT). A + T content was 65.39% (A: 29.27%, C: 16.59%, G: 18.03%, and T: 36.12%). ATG was the start codon in 11 PCGs: *COX1*, *COX2*, *ATP8*, *ATP6*, *COX3*, *NAD2*, *NAD6*, *NAD4l*, *NAD1*, *NAD4*, and *COB*. *NAD5* and *NAD3* had GTG as the start codon. TAG was the stop codon for *COX2*, *NAD6*, and *COB*. The other 10 PCGs were terminated by TAA. The neighbor-joining phylogenetic tree of the 15 related jellyfish species showed that *A. flagellatus* is closely related to *Nemopilema nomurai* and *Rhopilema esculentum*.

In recent decades, jellyfish blooms have frequently appeared in coastal seas worldwide (Condon et al. 2013). Jellyfish blooms cause serious damage to coastal economic development and marine ecosystem, and pose a challenge to human safety (Purcell 2007; Richardson et al. 2009). Extensive studies have examined the mechanisms of jellyfish blooms by focusing on understanding the eco-physiological characteristics of their life cycle stages (Lucas et al. 2012; Purcell 2012; Pitt et al. 2018). However, few studies have focused on the molecular phylogenetic relationships among jellyfish species based on their mitochondrial genomes (Zou et al. 2012; Hwang et al. 2014; Feng et al. 2019). This is important as it may provide insight into the similarity between different species of jellyfish.

The scyphozoan *Acromitus flagellatus* (Maas, 1903) belongs to Catostylidae (Family) under the Suborder Dactyliophorae. In recent years, medusae of *A. flagellatus* appeared in large numbers in the coastal waters of Hainan Province, China, from April to June (Du et al. 2021). To determine the molecular phylogenetic relationships between *A. flagellatus* and other jellyfish, its complete mitochondrial DNA was sequenced. An *A. flagellatus* medusa was collected from the eastern coast of Hainan Province (19.57° N, 110.83° E) and frozen in an ice box containing dry ice. The sample was then transferred to a −80 °C freezer at the Institute of Oceanology, Chinese Academy of Sciences (http://english.qdio.cas.cn/, Song Feng: fengsong@qdio.ac.cn) under the voucher ‘*A. flagellatus* ①.’ A piece of muscle tissue from *A. flagellatus* medusa was used for extraction of mitochondrial DNA. The extracted DNA was also stored in a −80 °C freezer, as described above, for sequencing (voucher: DNA-*A. flagellatus* ①).

The complete mitochondrial genome of *A. flagellatus* showed a linear molecular structure with a length of 16,779 bp (GenBank accession no. OM457248.1). There were 13 protein-coding genes (PCGs), small and large subunit ribosomal RNAs (s-rRNA and l-rRNA), and methionine and tryptophan transfer RNAs (trna-W-TCA and trna-M-CAT) in the mitochondrial genome. The base composition was 29.27%, 18.03%, 16.59%, and 36.12% for A, G, C, and T, respectively. A + T base composition was less than 70% (65.39%), similar to that in *Nemopilema nomurai and Rhopilema esculentum* (Wang and Sun 2017a, 2017b).

Of the 13 PCGs in *A. flagellatus* mitochondrial genome, 11 started with ATG (*COX1*, *COX2*, *ATP8*, *ATP6*, *COX3*, *NAD2*, *NAD6*, *NAD4l*, *NAD1*, *NAD4*, and *COB*). Only *NAD5* and *NAD3* had GTG as the start codon. Complete stop codons were present in all the genes. TAG was the stop codon for *COX2*, *NAD6*, and *COB*. The other 10 PCGs terminated with TAA as the stop codon. The neighbor-joining phylogenetic tree of 15 jellyfish species constructed using the complete mitochondrial genome from NCBI (Figure 1), showed that *A. flagellatus* was closely related to *N. nomurai* (GenBank no. KY454767.1) and *R. esculentum* (GenBank no. NC_035741.1).

**Figure 1.**
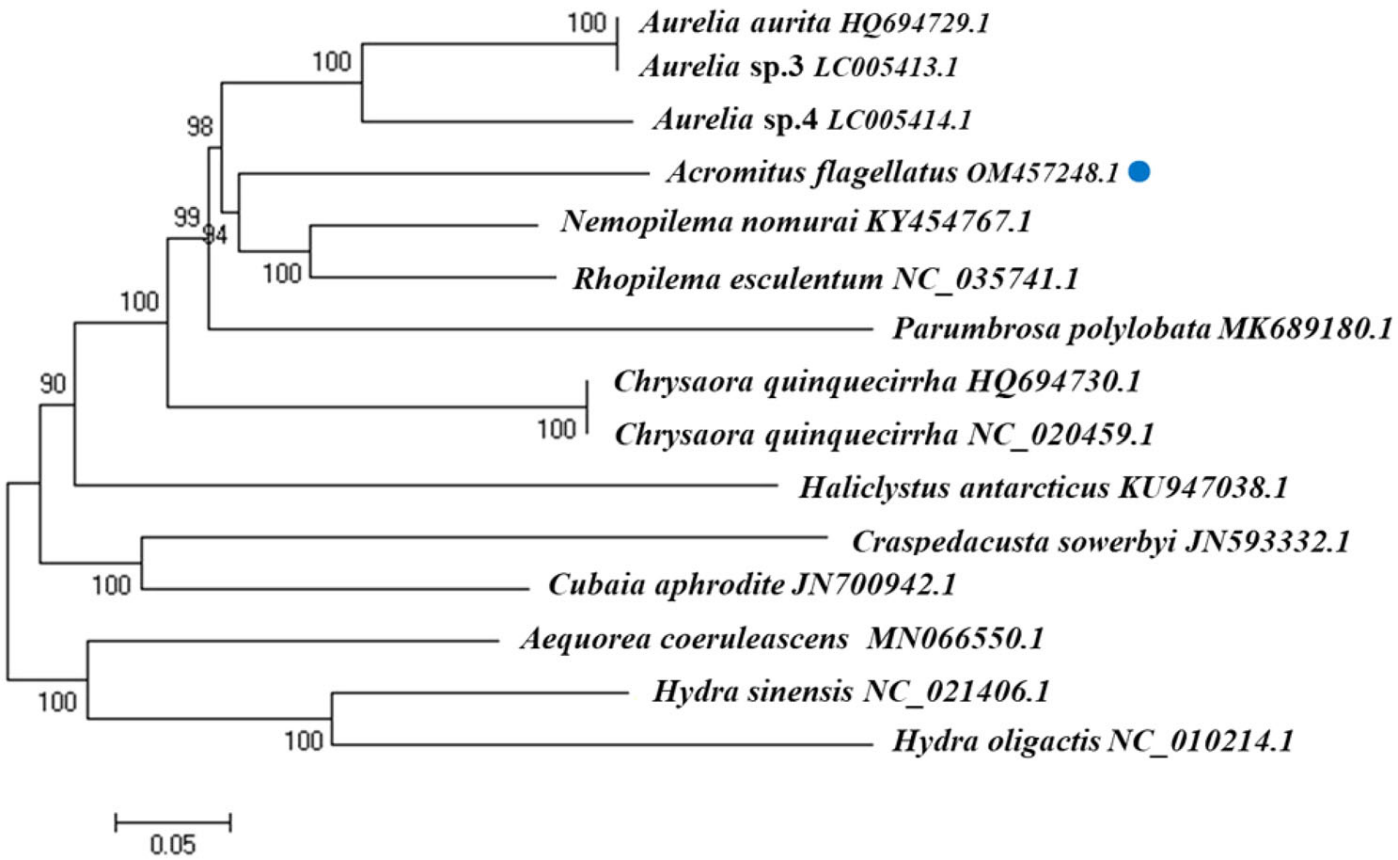
Phylogenetic relationship revealed by neighbor-joining tree.

## Data Availability

The genome sequence data that support the findings of this study are openly available in GenBank (https://www.ncbi.nlm.nih.gov/) accession no. OM457248.1. The associated BioProject, SRA, and Bio-Sample numbers were PRJNA817707, SRR18358023, and SAMN26806304, respectively.
